# Inhibitory effect of benzocaine from *Schisandra chinensis* on *Alternaria alternata*

**DOI:** 10.1038/s41598-024-57237-1

**Published:** 2024-03-20

**Authors:** Lin Fang Long, Qi Fang Zhao, Fu Long Zhang, Ran Tang, Jia Bao Wei, Shan Guan, Yan Chen

**Affiliations:** 1 Key Laboratory of Urban Agriculture in North China, Ministry of Agriculture and Rural Affairs, P. R. China, College of Bioscience and Resources Environment, Beijing University of Agriculture, Beijing, 102206 China; 2 Inner Mongolia Kingbo Biotechnology Co., Ltd., Bayannur, 015200 Inner Mongolia China

**Keywords:** Plant sciences, Gene expression profiling, Antifungal agents

## Abstract

The clinical effects of *Schisandra chinensis* against human disease are well-documented; however, studies on its application in controlling plant pathogens are limited. Here, we investigated its inhibitory effect on the growth of *Alternaria alternata*, a fungus which causes significant post-harvest losses on apples, known as black spot disease. *S. chinensis* fruit extract exhibited strong inhibitory effects on the growth of *A. alternata* with an EC_50_ of 1882.00 mg/L. There were 157 compounds identified in the extract by high performance liquid chromatography-mass spectrometry, where benzocaine constituted 14.19% of the extract. Antifungal experiments showed that the inhibitory activity of benzocaine on *A. alternata* was 43.77-fold higher than the crude extract. The application of benzocaine before and after *A. alternata* inoculation on apples prevented the pathogen infection and led to mycelial distortion according to scanning electron microscopy. Transcriptome analysis revealed that there were 4226 genes differentially expressed between treated and untreated *A. alternata*-infected apples with benzocaine. Metabolomics analysis led to the identification of 155 metabolites. Correlation analysis between the transcriptome and metabolome revealed that benzocaine may inhibit *A. alternata* growth via the beta-alanine metabolic pathway*.* Overall, *S. chinensis* extract and benzocaine are environmentally friendly plant-based fungicides with potential to control *A. alternata*.

## Introduction

Apples have gained immense popularity among consumers owing to their abundant nutrition and unique flavour, leading to extensive cultivation and consumption^1,2^. The total acreage dedicated to apple cultivation and production output rank among the highest worldwide. In 2021, the apple planting area and production scale in China exceeded 50% of the global aggregate^3^.

However, various apple diseases manifest with distinct symptoms at different stages, affecting the quality and yield of the produce to a certain extent because of inadequate scientific planting techniques and management, as well as adverse climatic conditions. Black spot is a major postharvest infectious disease of apples caused by *Alternaria alternata*^4^. This condition leads to fruit decay, characterised by a dense covering of black or dark green mould on the affected area, often accompanied by white floccules adhering to the mould layer^5^. These manifestations can severely affect the market value of the fruit, which can result in economic losses of over 50%^6^. The incidence rate of black spot on apple is higher in the late picking stage owing to fruit senescence and the consequent reduction in inherent disease resistance^7^. Therefore, effective control measures for black spot disease are pivotal to improving the postharvest quality of apples.

Schisandraceae (Magnoliidae) is a family of flowering plants characterised by perennial deciduous vines^8^. It encompasses approximately 39 species distributed non-continuously across Southeast Asia and North America^9^. Among them, *Schisandra chinensis* (Turcz.) Baill. is particularly rich in various chemical components, including lignans, volatile oils, polysaccharides, organic acids, terpenes, and flavonoids^10^, many of which exhibit pharmacological effects. Modern pharmacological studies have established that *S. chinensis* fruits exhibit therapeutic effects, including sedation, hypnosis, liver protection, and antitumour properties, together with efficacy in treating cough and asthma^11,12^. Therefore, *S. chinensis* holds medicinal value in traditional Chinese medicine. The main antifungal components of *S. chinensis* are lignans, particularly schisandrin, schisanterin A, and deoxyschizandrin; the minimum inhibitory concentration of *S. chinensis* ethanol extract against *Staphylococcus aureus* and *Mucor*, *Escherichia coli,* and *Aspergillus* was 1.25 mg/mL, 6.25 mg/mL, and 0.625 mg/mL, respectively^13^. Gomisin J in *S. chinensis* fruit exhibits anticancer activity, with a low concentration (30 μg/mL) inhibiting cell proliferation and reducing cell survival, and induces programmed cell necrosis and apoptosis^14^. Moreover, biflavones identified in *Schinus terebinthifolius* fruits exhibit antifungal activity. Among them, tetrahydroamentoflavone has a significant impact on the formation and composition of biofilms^15^. Plant-derived flavonoids are able to compromise the integrity and permeability of cell wall membranes, in turn destroying the cellular morphology of microorganisms^16^. These studies indicate that *S. chinensis* has several chemical components with activities against various pathogenic fungi, suggesting its potential in controlling black spot on apple. Given its status as a traditional Chinese medicine plant, exploring the effective antifungal components of *S. chinensis* provides the possibility to develop new botanical pesticides, thereby reducing the negative impact associated with chemical pesticides.

Benzocaine (also known as ethyl p-amino benzoate, with the chemical formula C_9_H_11_NO_2_)^17^ is an *S. chinensis*-derived phenylpropanoid that exhibits low solubility in water but is readily soluble in organic solvents, such as alcohols, ethers, and chloroform^18^.It is widely used as a local anaesthetic, and exhibits clinical application in analgesia and antipruritic treatment of wounds, ulcers, burns, skin abrasions, and haemorrhoids^19–22^. It has antifungal, anti-inflammatory, and anticancer properties^23^, rendering it a widely used medicinal compound. Benzocaine gel has demonstrated efficacy in treating oral ulcers and toothaches; it also has antifungal activity against *S. aureus*^24^. However, there is a dearth of research on the application of benzocaine in controlling plant pathogens, underscoring the importance of this study.

This study investigated the antifungal activity and the preliminary mechanism of *S. chinensis* fruit extract against *A. alternata*. We also aimed to assess its main antifungal components, providing theoretical and technical support to prevent the occurrence of black spot on apple postharvest and during storage.

## Results

### Effects of *S. chinensis* fruit extract on *A. alternata* growth in vitro

The active components in *S. chinensis* were extracted using 75% ethanol. Spinning and steaming 100 g of the crude extract of *S. chinensis* fruit yielded 33.88 g.

**Figure 1:**
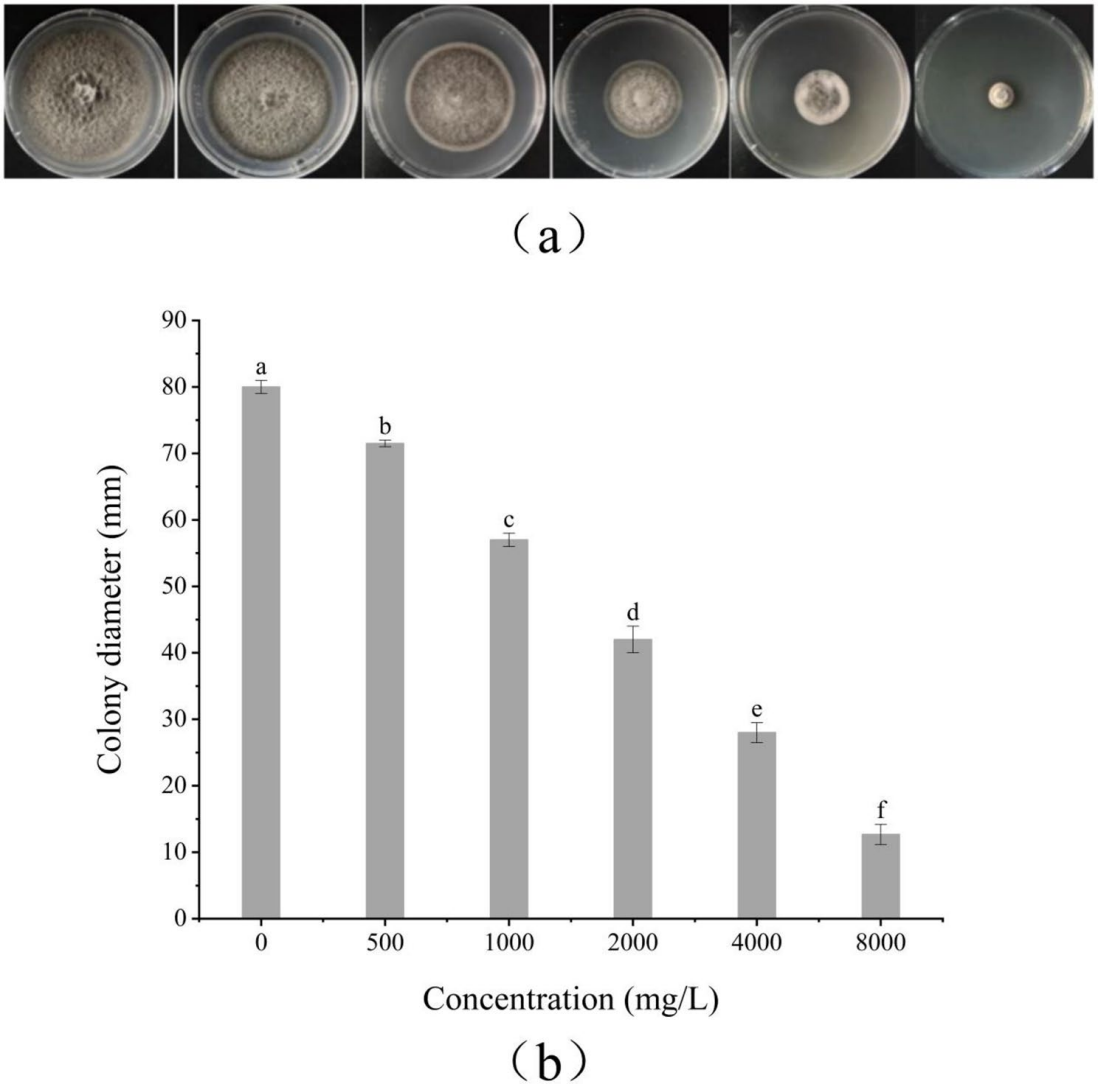
In vitro antifungal activities of *S. chinensis* fruit extract against mycelial growth of *A. alternata* after 3 days incubation at 28 ± 1 ℃. (**a**) The inhibitory effect of *S. chinensis* fruit extract on *A. alternata;* the concentration of *S. chinensis* fruit extract from left to right is 0; 500; 1000; 2000; 4000 and 8000 mg/L. 0 is solvent. (**b**) Indicating the colony diameter with treatment with *S. chinensis* fruit extract. Data are presented the means of the average data. Error bars represent the standard deviation (SD) of the means (n = 3). The bars with different letters indicate significant differences at P < 0.05 according to Duncan’s multiple range test comparisons. (Toxicity regression equation, y = 2.0674x + 4.4323; EC_50_, 1,882.00 mg/L; details are given in Supplemental Data, Table S1, Figure S1).

The inhibitory effect of *S. chinensis* fruit extract on *A. alternata* was assessed using the mycelial growth rate method. *S. chinensis* fruit extract showed concentration-dependent inhibition against *A. alternata* (Fig. 1). At a concentration of 8000 mg/L, *S. chinensis* fruit extract demonstrated an inhibitory rate of 91.9% against *A. alternata*, with a 50% maximum effective concentration (EC_50_) value of 1882.00 mg/L in vitro*.* We obtained the regression equation of toxicity as y = 2.0674x + 4.4323 after considering the concentration logarithm value of the *S. chinensis* fruit extract as the abscissa, and the probability value inhibition rate of the colony diameter as the ordinate (Supplemental Table S1, Figure S1).

### Identified compounds in *S. chinensis* extract

**Figure 2:**
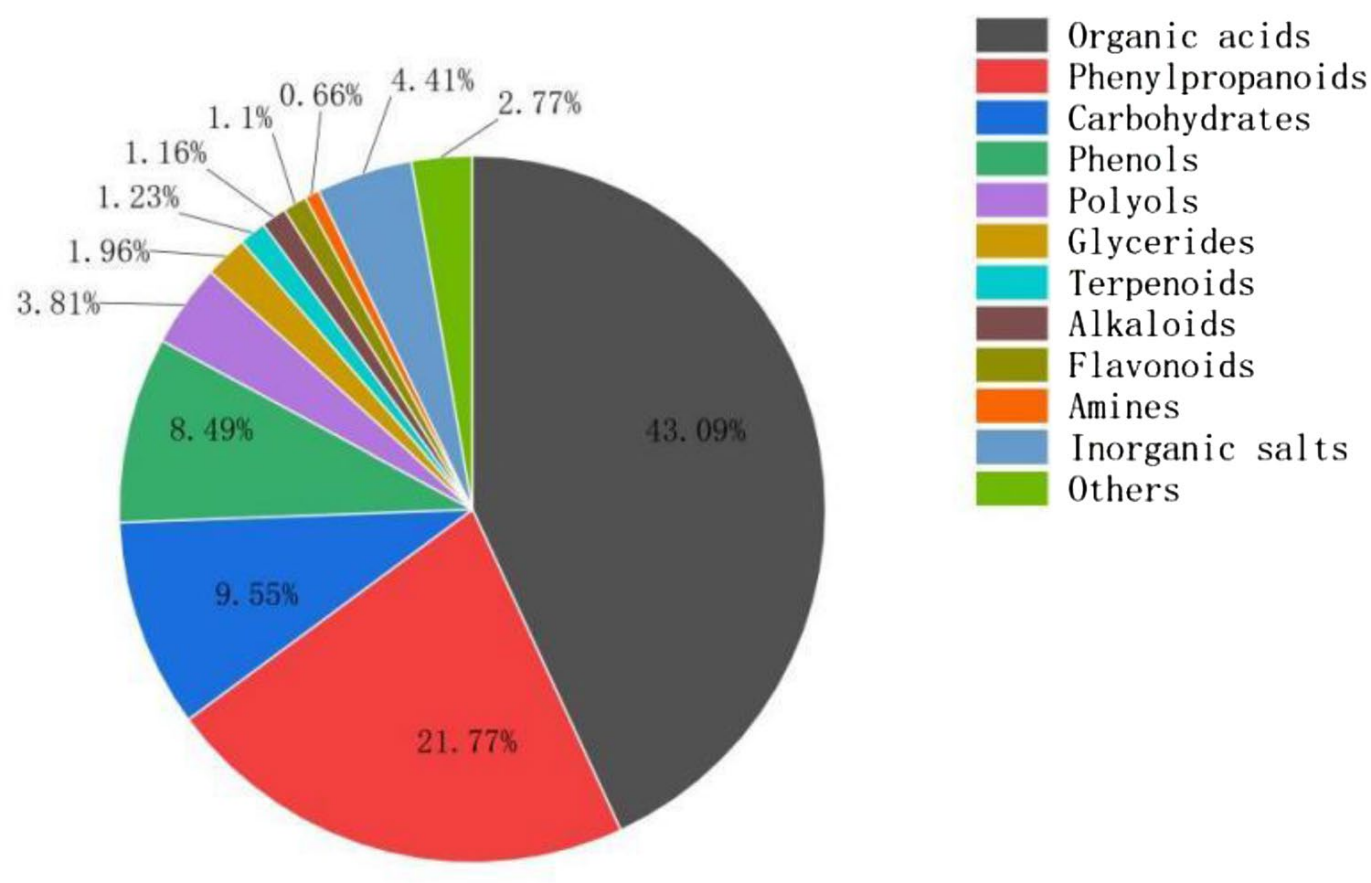
The identified compounds in the extract of *S. chinensis* fruit.

A total of 157 compounds were detected in the fruit extract of *S. chinensis* using liquid chromatography-mass spectrometry (LC/MS). These compounds can be roughly characterised into 11 categories. Among them, the content of organic acids was the highest, comprising 56 categories and constituting 43.09% of the total detected amount, followed by phenylpropanoids (6 categories, 21.77%), carbohydrates (12 categories, 9.55%), and phenolic compounds (8.49%) (Fig. 2). In addition to these compounds, the extract also contained phenols, polyols, flavonoids, alkaloids, glycerol esters, amines, terpenes, and inorganic salts.

Twenty-two compounds accounted for over 1% of the total components (Supplemental Data, Table S2), encompassing two, categories with pelargonic acid and benzocaine accounting for over 10%, constituting 18.93% and 14.19% of the total detected amount, respectively.

Compound testing results revealed that the relative contents of organic acids and phenylpropanoids were relatively high, regardless of the type or relative content of the compound. *S. chinensis* extract exhibited an inhibitory effect on *A. alternata*; therefore, the probability of the aforementioned compounds having inhibitory activity on the fungus was considered high. We compared the inhibitory effects of various *S. chinensis* constituents on *A. alternata* and found that benzocaine exhibited the best inhibitory effect.

### Antifungal activity of benzocaine

**Figure 3:**
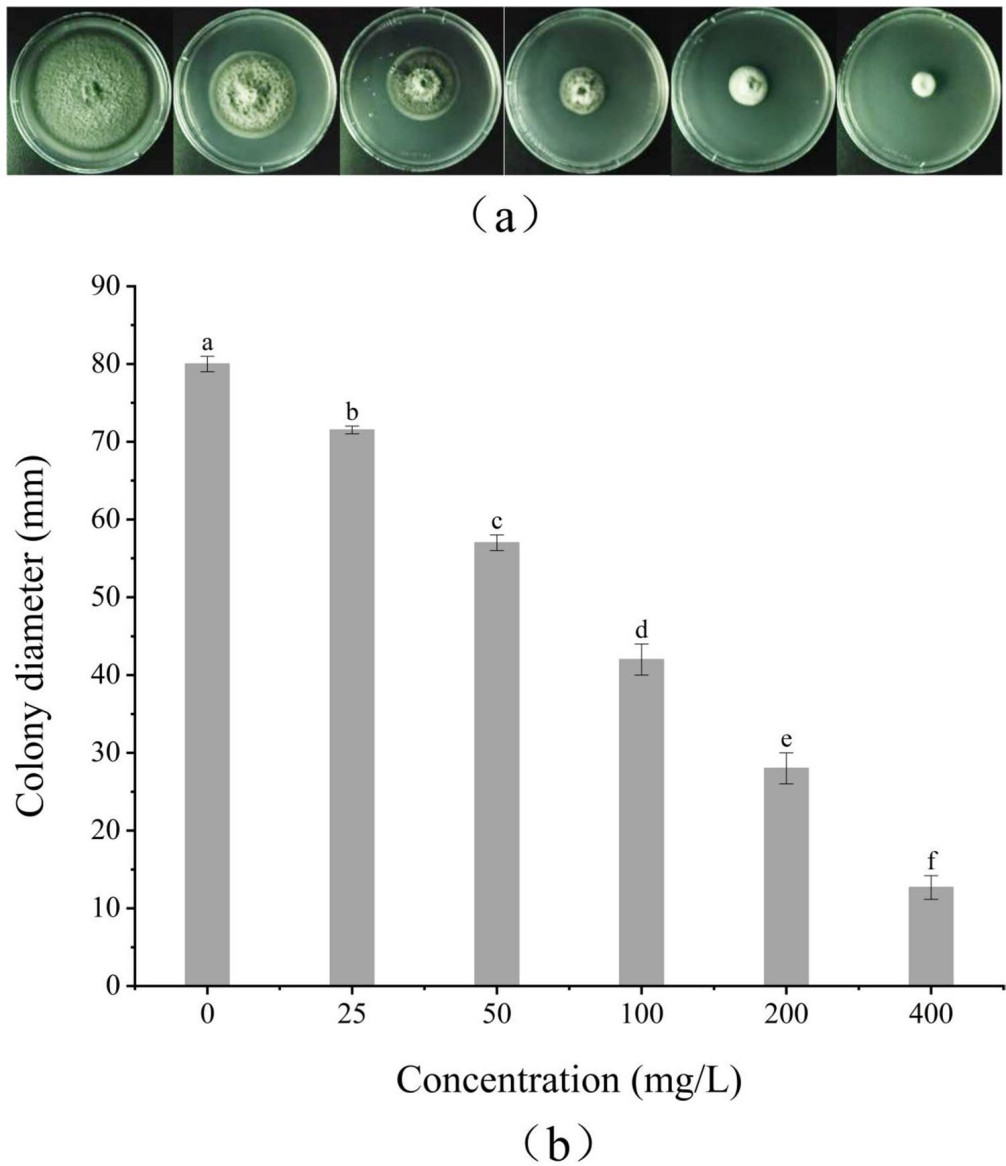
In vitro antifungal activities of benzocaine against mycelial growth of *A. alternata* after 3 d incubation at 28 ± 1 ℃. (**a**) The inhibitory effect of benzocaine on *A. alternata*; The concentration of benzocaine from left to right is 0; 25; 50; 100; 200 and 400 mg/L. 0 is solvent. (**b**) Indicating the colony diameter with treatment with benzocaine. Data are presented the means of the average data. Error bars represent the SD of the means (n = 3). The bars with different letters indicate significant differences at P < 0.05 according to Duncan’s multiple range test comparisons. (Toxicity regression equation, y = 1.2652x + 6.7291; EC_50_, 42.99 mg/L; details are given in Supplemental Table S3).

The mycelial growth rate method was employed to detect the inhibitory effect of benzocaine against *A. alternata* (Table S3: toxicity regression equation, y = 1.2652x + 6.7291; EC_50_, 42.99 mg/L). An increase in the benzocaine concentration resulted in a corresponding increase in its inhibitory effect against *A. alternata,* showing a significant linear relationship (Fig. 3). Moreover, the inhibitory effect of benzocaine on *A. alternata* was significantly higher (43.77-fold) than that of *S. chinensis* fruit extract.

### Effect of benzocaine on the growth of *A. alternata *in vivo

**Figure 4:**
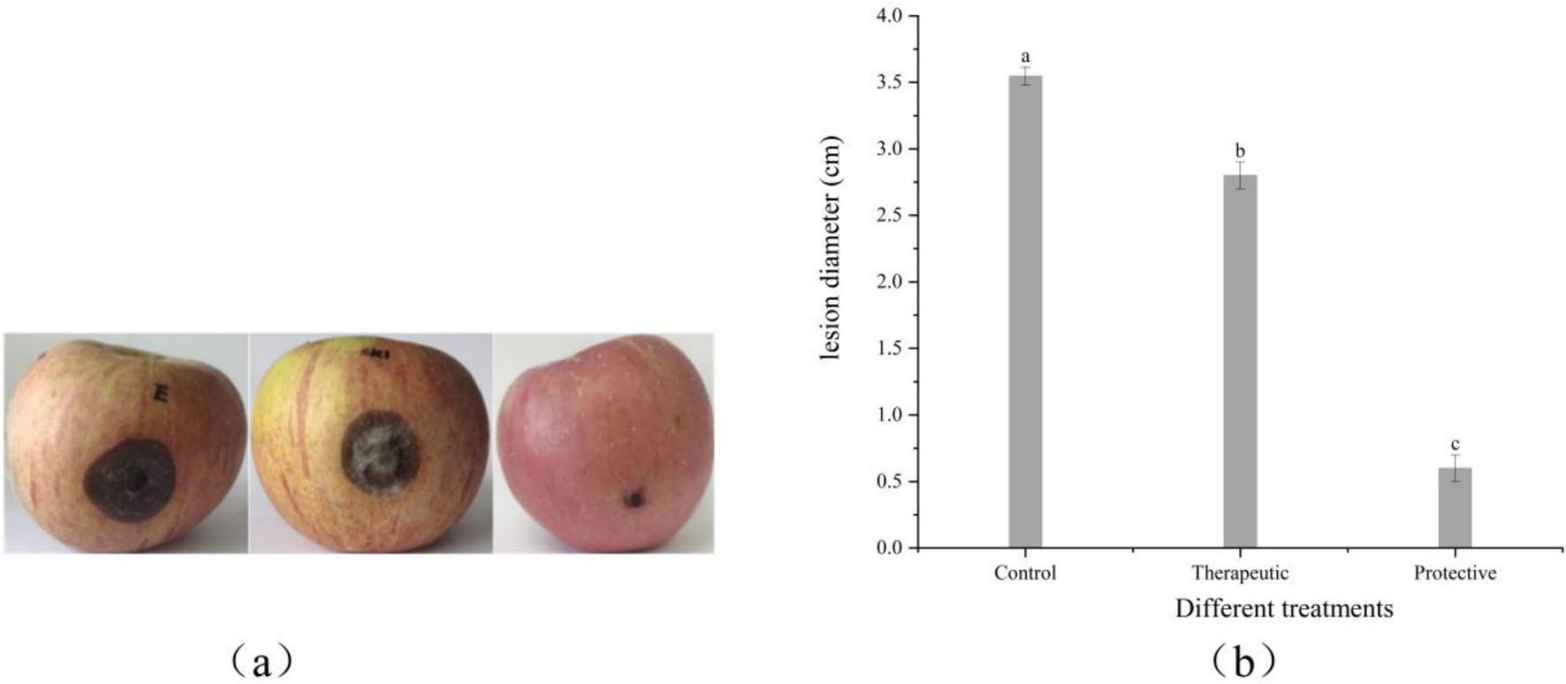
Effects of benzocaine on black spot on apple after a 7 days post-inoculation with *A. alternata* at 28 ± 1 ℃. (**a**) The treatment of benzocaine from left to right control; therapeutic (42.99 mg/L); protective (42.99 mg/L). Control is solvent. (**b**) Indicating the lesion diameter with treatment with benzocaine. Data presented are the means of the average data. Error bars represent the SD of the means (n = 3). The bars with different letters indicate significant differences at P < 0.05 according to Duncan’s multiple range test comparisons.

Seven days post-inoculation with *A. alternata* (Fig. 4), apples untreated with benzocaine displayed disease spread, resulting in a disease spot diameter of 3.55 cm. Post-inoculation with *A. alternata* followed by benzocaine application before the onset of apple disease yielded an average lesion diameter of 2.8 cm, indicating the therapeutic effect of benzocaine against black spot on apple, with an efficacy of 21.13%. Conversely, the average lesion diameter was 0.6 cm when 50 µL (42.99 mg/L) of benzocaine was sprayed before inoculation with *A. alternata*, indicating the protective effect of benzocaine against black spot on apple, with an efficacy of 83.1%. Therefore, benzocaine demonstrated efficacy in preventing the occurrence of black spot and exhibited certain therapeutic effects on black spot on apple.

### Effect of benzocaine on *A. alternata* mycelium

**Figure 5:**
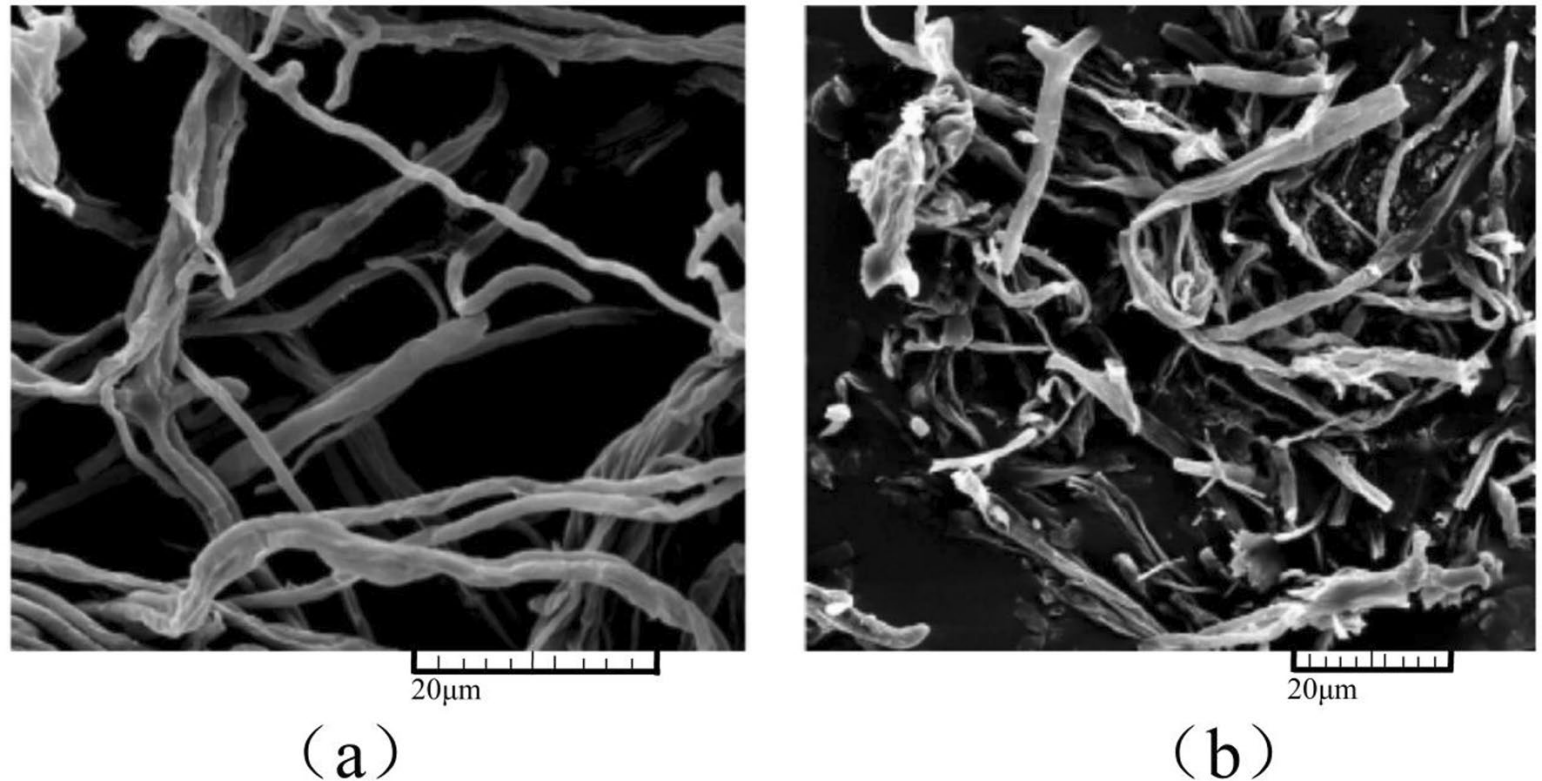
Scanning electron microscopy images of the morphology of *A. alternata* mycelium under different treatments. (**a**) Control; (**b**) Benzocaine treatment (42.99 mg/L). Control is solvent. Three biological repeats were conducted.

Scanning electron microscopy (SEM) results showed a significant difference between the treatment (EC_50_, 42.99 mg/L) and control groups. In the control group, the mycelia were elongated and plump with a regular morphology (Fig. 5a), which was significantly different from that in the treatment group (Fig. 5b). The surface of the mycelia in the treatment group was ruptured, with irregular morphology and fragmentation.

### Differential gene expression analysis in benzocaine- treated *A. alternata*

**Figure 6:**
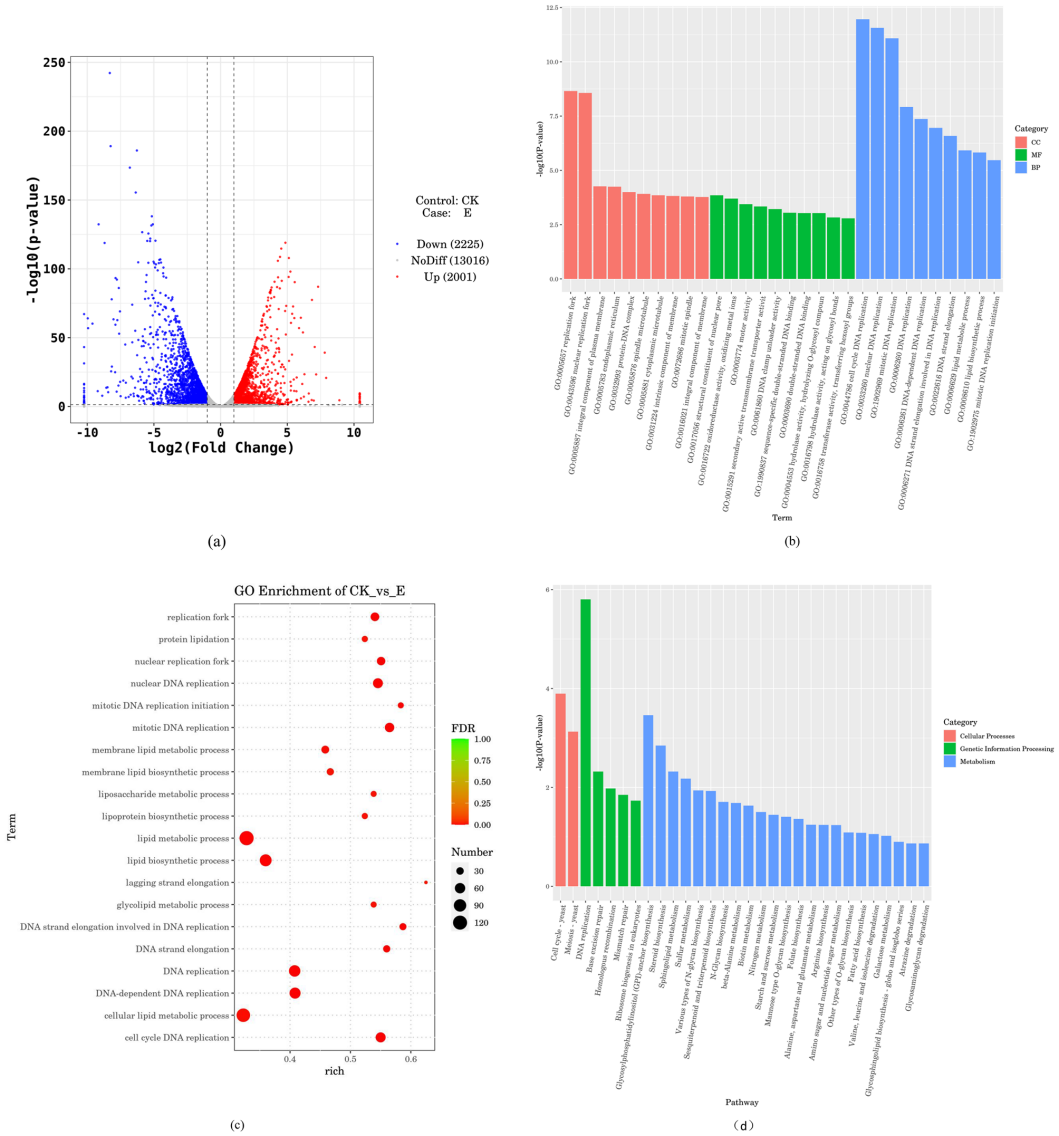

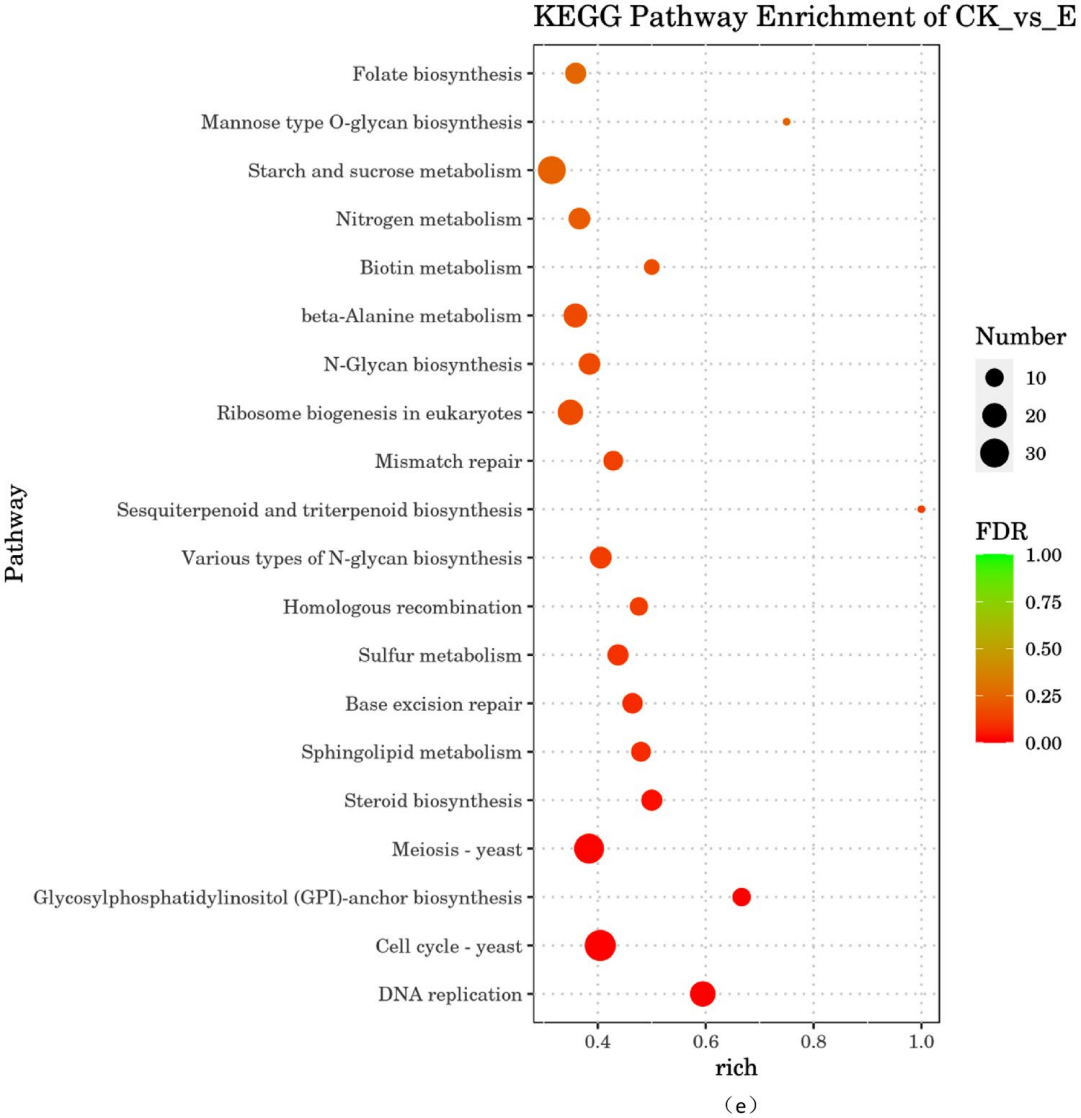
Transcriptome differential gene expression analysis of *A. alternata* after benzocaine treatments. (**a**) Volcano diagram of DEGs after benzocaine treatments compared to the control. (**b**) Top 10 pathways of GO functional classification analysis among treatment groups of DEGs. (**c**) Top 20 pathways of GO enrichment bubble shared by the treatment groups of DEGs. (**d**) Top 20 pathways of KEGG metabolic pathways classification among treatment groups of DEGs. (**e**) Top 20 pathways of KEGG enrichment bubble shared by the treatment groups of DEGs.

To further analyse the antifungal mechanism of benzocaine, we conducted transcriptomic analysis to assess its effect on *A. alternata*. The criteria of significant differential expression were | log_2_ (fold change) |≥ 1 and P-value ≤ 0.05. The transcriptome sequencing data from the control and benzocaine treatment groups were standardised and a volcano plot was generated to illustrate the gene expression patterns (Fig. 6a). The grey portion represents genes with insignificant differences, whereas the red and blue portions represent genes with significant upregulation and downregulation, respectively. After treatment with benzocaine, 2001 genes were upregulated, 2225 genes were downregulated, and 13,016 genes were not significantly differentially expressed genes (DEGs).

Gene Ontology (GO) analysis is a standardised classification system for gene functions, which provides strictly defined concepts and structured vocabulary for gene characteristics and products^30^. We performed GO term enrichment analysis of DEGs between the benzocaine treatment and control groups. The DEGs annotated with GO terms were used to calculate the gene list and the number of genes associated with each term. The hypergeometric distribution method was employed to calculate P-value and identify significantly enriched GO terms. The GO terms were classified into three categories: cellular components (CCs), molecular functions (MFs), and biological processes (BPs). The top 10 significantly enriched GO terms in each category are shown in Fig. 6b (P-value ≤ 0.05). There were notable differences in the expression of items related to various CCs, including replication forks, nuclear replication forks, plasma membrane components, and endoplasmic reticulum. DEGs were found in BPs such as cell cycle DNA replication, nuclear DNA replication, mitotic DNA replication, and DNA replication, indicating that benzocaine may affect *A. alternata* DNA replication*.* DEGs were observed in MFs such as structural components of nuclear pores, oxidoreductase activity, oxidative metal ions, sports activity, and secondary active transmembrane transport protein activity.

To measure the degree of enrichment, we used parameters such as rich factor, false discovery rate (FDR ≤ 0.05) values, and the number of genes enriched in a given GO term based on the GO enrichment results. Rich factor represents the ratio of the number of enriched differential genes to the number of annotated differential genes, while FDR is a P-value correction factor. Figure 6c displays the results; terms such as lagging strand elongation, mitotic DNA replication initiation, DNA strand extension in DNA replication, mitotic DNA replication, and DNA strand extension exhibited high rich factor values, indicating a significant degree of enrichment. The DEGs were concentrated in DNA replication. Other enriched terms included lipid metabolism process, cellular lipid metabolism process, lipid biosynthesis process, DNA replication, and DNA-dependent DNA replication, which involved many genes. This indicated that DEGs were concentrated in lipid metabolism and DNA replication processes.

Kyoto Encyclopedia of Genes and Genomes (KEGG) enrichment analysis was performed to investigate the effect of benzocaine on *A. alternata* using the KOBAS annotation system with the results mapped to the corresponding pathways^31^. The KEGG-enriched genes were categorised into five parts: metabolism, genetic information processing, environmental information processing, cellular processes, and organismal systems. Out of the top 20 pathways with P-value ≤ 0.05 (Fig. 6d), only two pathways related to cellular processes were highly significant: cell cycle-yeast and meiosis-yeast. Five pathways were related to genetic information processing, among which DNA replication had the highest significance. Twenty-three pathways were related to metabolism, with benzocaine affecting most metabolic pathways. The bubble diagram shows that sesquiter penoid and triterpenoid biosynthesis, mannose-type O-glycan biosynthesis, GPI anchor biosynthesis, and DNA replication exhibited high rich factor values, indicating a significant degree of enrichment (Fig. 6e; 0 < FDR > 1). Additionally, DEGs were abundant in pathways such as cell cycle-yeast, meiosis-yeast, DNA replication, starch and sucrose metabolism, and ribosomal biogenesis in eukaryotic cells.

### Validation results of fluorescence quantitative PCR

After conducting GO and KEGG enrichment analyses, eight DEGs with significant changes across different items and metabolic pathways were selected, and the expression results were validated using real-time fluorescence quantitative PCR (RT-qPCR) following reverse transcription. The comparison between the transcriptome and RT-qPCR results (Supplemental Data, Table S4) revealed a discernible difference in the log_2_FC (| log_2_FC |> 2) between the two methods. However, the trend of differential expression was consistent, indicating that the transcriptome data were reliable.

### Differential metabolome and pathway analysis

**Figure 7:**
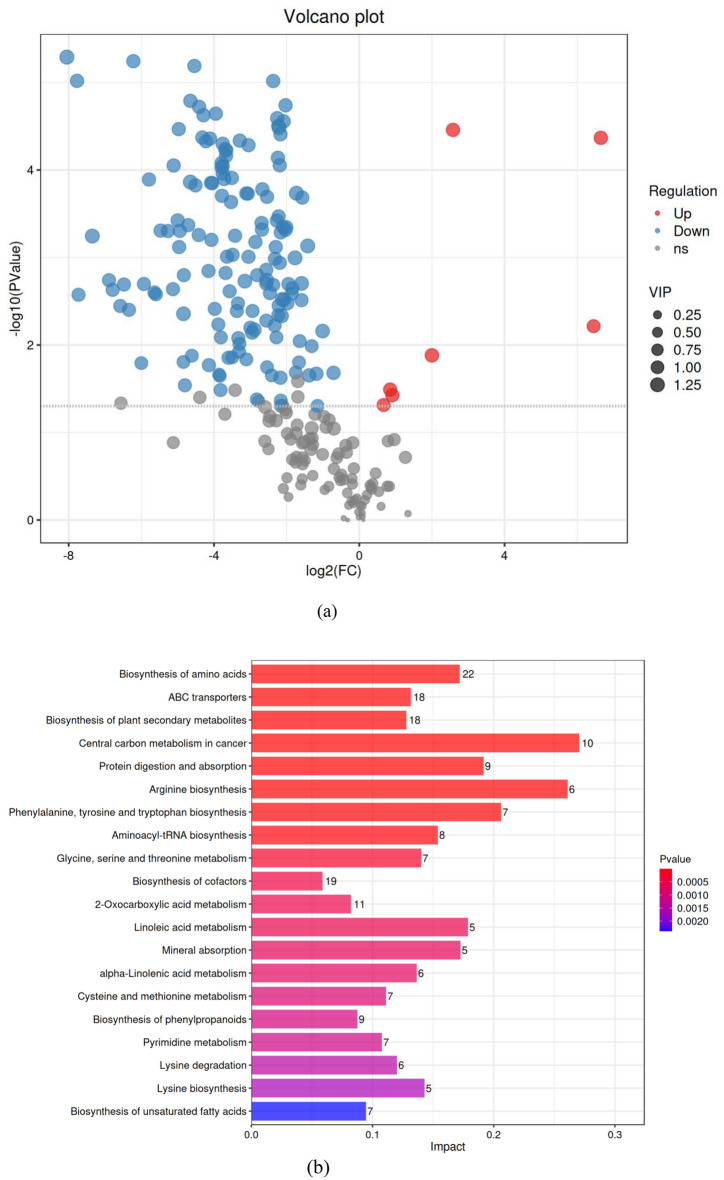
Volcano diagram and KEGG enrichment bar chart. (**a**) Volcano diagram of differential metabolites after benzocaine treatments compared to the control (Each point in the graph represents a metabolite, and the horizontal axis represents the logarithmic value of Log_2_, which is the quantitative difference multiple of a metabolite between the two samples; the y-axis represents the logarithmic value of − log_10_ for the P-value. The larger the absolute value of the horizontal axis, the greater the multiple difference in the expression level of a certain metabolite between the two samples; the larger the vertical axis value, the more significant the differential expression, and the more reliable the screened differential expression metabolites). (**b**) KEGG enrichment bar chart. analysis of differential metabolites after the action of benzocaine (the vertical axis represents metabolic pathways, and the horizontal axis represents the impact values enriched in different metabolic pathways. This value can be simply understood as contribution, that is, the higher the value, the higher the contribution of metabolites detected in this pathway, and the colour is related to the P-value).

To further understand the antifungal mechanism of benzocaine, an analysis of its impact on the metabolome of apple black spot pathogens was conducted based on transcriptomics (Fig. 7a (FC ≥ 2 or ≤ 0.5 and VIP ≥ 1)), indicating 148 downregulated metabolites after treatment (blue section), 7 upregulated metabolites (red section), and 91 metabolites with insignificant differences (grey section).

A total of 155 differential metabolites were analysed following KEGG enrichment and the top 20 metabolic pathways are shown in Fig. 7b (P-valve ≤ 0.05). The most significant pathway with the highest impact value was central carbon metabolism in cancer, followed by arginine biosynthesis, phenylalanine, tyrosine and tryptophan biosynthesis. This suggested that differential metabolites exert the most significant influence on these three pathways. Differential metabolites also have a noteworthy impact on several other pathways, including protein digestion and absorption, biosynthesis of amino acids, ABC transport, biosynthesis of plant secondary metabolites, and aminoacyl-tRNA biosynthesis.

### Combined analysis of transcription and metabolome

The correlation between DEGs and differential metabolites were explored based on transcriptome and metabolome data to further clarify the mechanism of action of benzocaine against *A. alternata*.

**Figure 8:**
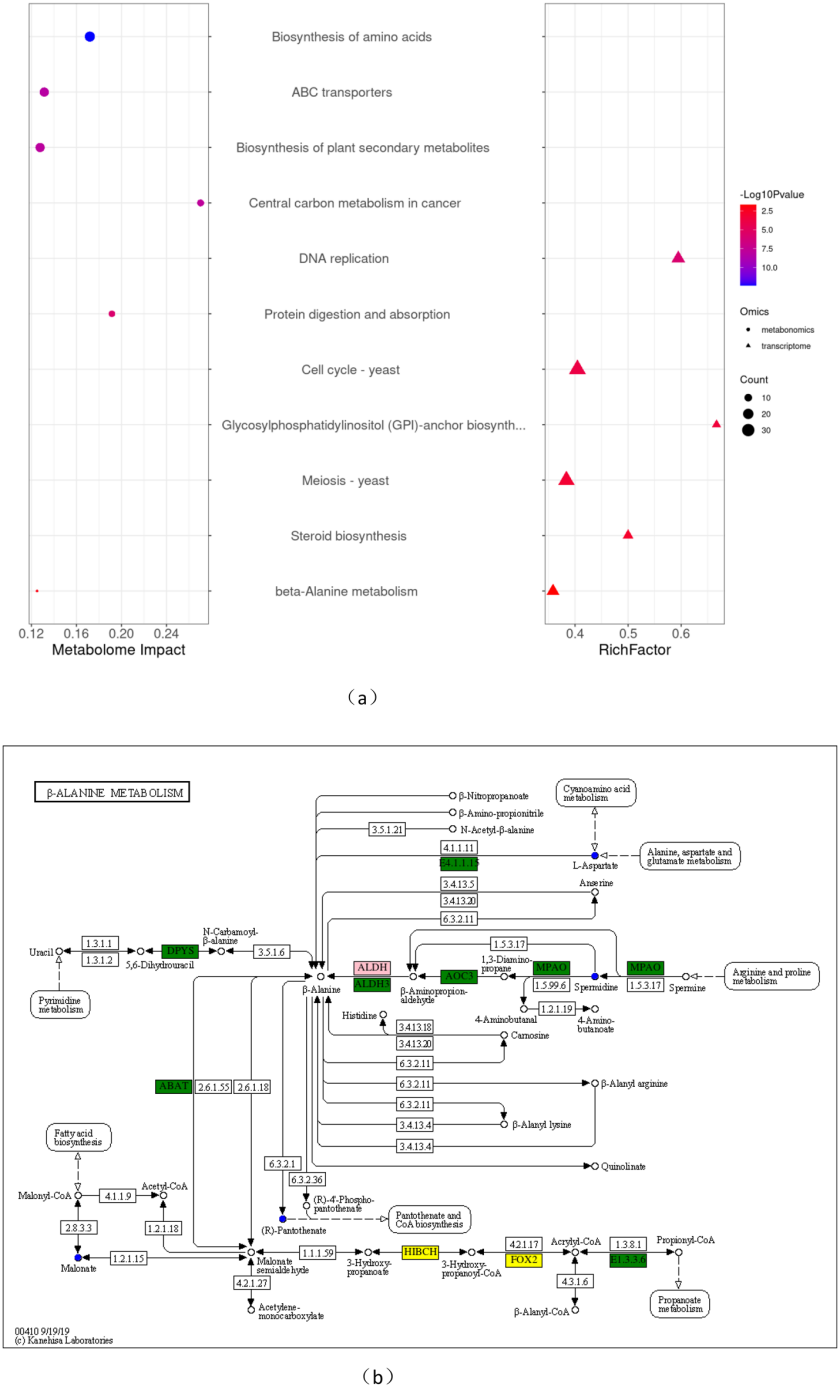
Combined analysis of transcription and metabolome and pathways of beta-alanine metabolism of KEGG. (**a**) The bubble on the left represents the KEGG pathway of DEGs in the metabolome, while the triangle on the right represents the KEGG pathway of DEGs in the transcriptome. (**b**) Pathway of beta-alanine metabolism in KEGG (blue circles indicate downregulated differential metabolites; the yellow box represents upregulated genes, the green box represents downregulated genes, and the pink box represents DEGs).

The results revealed 50 enriched KEGG pathways in the metabolome and 20 in the transcriptome (P-value ≤ 0.05). Multi-omics association analysis found that the only shared metabolic pathway between the metabolome and transcriptome was the beta-alanine metabolism pathway (Fig. 8a). This indicates that benzocaine may affect the metabolism of *A. alternata* through this pathway. The differentially downregulated metabolites involved in this pathway include L-aspartic acid, spermidine, malonate, and pantothenic acid; there were 19 DEGs, of which 5 were upregulated, including *HIBCH* and *FOX2*; there were 14 downregulated DEGs, including *DPYS, ABAT, ALDH3, AOC3, MPAO,* etc. (Fig. 8b).

## Discussion

The clinical effects of *S. chinensis* against human diseases are well-documented^32^. However, there is limited research on the use of its active substances in the formulation of botanical pesticides. Similary, the potential effectiveness of these active substances in inhibiting the growth of pathogenic fungi has not been determined. Therefore, we focused on the components responsible for inhibiting *A. alternata*, after selected *S. chinensis* as a plant source material, to identify plant-derived ingredients that effectively control black spot on apple.

The inhibitory effect of the petroleum ether segment on *A. alternata* was significantly weaker than the ethyl acetate and n-butanol segments. Therefore, we utilised an approach where effective components were first extracted using ethanol, followed by petroleum ether to remove compounds with lower polarity. The inhibitory experiments further confirmed the superior effect of the extract obtained using this extraction method over ethyl acetate, n-butanol, and ethanol extracts.

The fruit extract of *S. chinensis* exhibited antifungal activity against *A. alternata*, with an inhibitory rate of 91.9% at a concentration of 8000 mg/L liquid chromatography-mass spectrometry analysis revealed the presence of organic acid and phenylpropanoid compounds in the *S. chinensis* fruit extract. Among them, the phenylpropanoid compound benzocaine demonstrated a strong inhibitory effect against *A. alternata*, with an EC_50_ of 42.99 mg/L. Benzocaine accounted for 14.19% of the extract, emerging as the main antifungal component. It prevented the occurrence of black spot on apple and exhibited certain therapeutic effects, primarily through its protective effects. The antifungal mechanism of benzocaine against *A. alternata* was further assessed using SEM and transcriptomics.

The surface of *A. alternata* hyphae treated with benzocaine was ruptured and had irregular morphology and fragmentation. Treatment with benzocaine resulted in the upregulation of 2001 genes and downregulation of 2225 genes in *A. alternata*. Real-time fluorescence quantitative PCR was used to validate the expression of eight DEGs. The data were consistent with the trend of transcriptome expression, demonstrating the reliability of the transcriptome data. Furthermore, GO and KEGG enrichment analyses revealed notable changes following treatment with benzocaine.

The results the transcriptome GO enrichment analysis showed that DEGs were enriched in 572 GO terms, including 375 terms involved in BPs, 100 terms involved in MFs, and 97 terms involved in CCs (P-value ≤ 0.05). The functions with significant differences were concentrated in terms related to DNA replication and membrane components.

KEGG analysis of DEGs after benzocaine treatment revealed 118 metabolic pathways, with 20 pathways identified as being significant (P-value ≤ 0.05). Among them, the pathway involved in genetic information processing had the highest significance, with five pathways associated with DNA replication and repair. There were two pathways associated with cellular processes (cell growth and death). There were 13 pathways involved in the metabolism, of glycan, lipids (compounds), amino acids, and vitamins. Enrichment analysis of DEGs in the transcriptome found that the effects of benzocaine on *A. alternata* were multifaceted and participated in multiple metabolic pathways. Further experimental evidence is needed to determine whether benzocaine directly affects DNA replication and other functions.

Analysis of the changes in *A. alternata* metabolites after treatment with benzocaine showed a total of 161 metabolic pathways following KEGG enrichment analysis, 50 of which were significant (P-value ≤ 0.05), including biosynthesis of amino acid, ABC transport, biosynthesis of plant secondary metabolites, central carbon metabolism in cancer, protein digestion and absorption, and amino acyl-tRNA biosynthesis, etc.

We analysed correlations between DEGs and differential metabolites to further clarify the correlation between transcriptome and metabolome data. We conducted association analysis on DEGs and differential metabolites involved in the same metabolic pathway through KEGG analysis. Multi-omics association analysis of KEGG pathways revealed 50 pathways in the metabolome and 20 in the transcriptome (P-value ≤ 0.05). Only the beta-alanine metabolism pathway was shared, indicating that benzocaine may inhibit the growth of *A. alternata* through this pathway. The differentially downregulated metabolites involved in this pathway included L-aspartic acid, spermidine, malonate, and pantothenic acid. There were 19 DEGs, of which 5 were upregulated, including *HIBCH* and *FOX2,* while 14 were downregulated, including *DPYS, ABAT, ALDH3, AOC3, MPAO,* etc. On this basis, further validation using techniques such as gene knockout is required to explain the target and mode of action of benzocaine.

In summary, this study found that the extract obtained from *S. chinensis* fruit extracted through organic solvent extraction has an inhibitory effect on fungi. The extract contained at least 157 compounds. Benzocaine accounted for 14.19% of the total extract and exhibited inhibitory activity against *A. alternata* (43.77-fold higher than that of the extract). Transcriptome and metabolomic analyses preliminarily proved the pathway of action of benzocaine. This article provides experimental evidence to pave the way for the development of new environmentally friendly plant-based fungicides using *S. chinensis* extract and benzocaine.

## Methods

### Strains, materials, and reagents

#### Plant ethics

The plant material used in this study was northern *S. chinensis* collected from Kuandian County, Liaoning Province, China (124.8° E; 40.8° N) (http://www.hywwz.cn). The fruits, leaves, and rattan stems were harvested in August to September, June, and October, respectively, and all samples were dried before use. The experimental material was obtained from a cultivated species and did not involve any relevant laws or regulations. The collection of plant material and all experimental research complied with institutional, national, and international guidelines and legislation. Permission to use *S. chinensis* seeds was obtained from the relevant institutions at the time of collection, and the material was solely used for this study.

The *A. alternata* strains were isolated from rotten apples and identified at Beijing University of Agriculture. Fuji apples were purchased from Beinong supermarket. The experimental equipment used in this study included an electrically heated constant temperature blast drying oven purchased from Shanghai Longyue Instrument Equipment Co., Shanghai, China; a multifunctional pulveriser purchased from Shanghai Bangy Industrial Co., Shanghai, China; a water bath purchased from Shanghai Boxun Industry & Commerce Co., Shanghai, China; and a tissue grinder purchased from Zhejiang Meibi Instrument Co., Zhejiang, China. The ultra-high-performance liquid phase system used in the study was purchased from Thermo Fisher Scientific, USA, and the chromatographic column was purchased from Waters Co., Milford, MA, USA.

Ethanol (purity ≥ 99.9%) and petroleum ether (purity ≥ 99.7%) were purchased from Tianjin Zhiyuan Chemical Reagent Co., Tianjin, China. Methanol (purity ≥ 99.0%) and acetonitrile (purity ≥ 99.9%) were purchased from Thermo Fisher Scientific Co., China. 2-Chloro-l-phenylalanine (purity 98%) was purchased from Aladdin Reagent Co., Shanghai, China. Formic acid (mass spectrometry-grade) was provided by Tihiai Chemical Industry Development Co., Shanghai, China. Ammonium formate (purity ≥ 99.9%) was purchased from Sigma Co., Shanghai, China. Benzocaine (analytically pure, 99% purity) was purchased from Shanghai McLean Biochemical Technology Co., Shanghai, China. Fungal RNA extraction kits were purchased from Omega Bio-Tek Co., USA. Phosphoric acid buffer solution was purchased from Beijing Aoqing Biotechnology Co., Beijing, China.

Freshly washed fruits were dried to a constant weight at 60 ℃ in an electrically heated constant temperature blast drying oven and subsequently pulverised into a fine powder using a multifunctional pulveriser to eliminate moisture content prior to the extraction of active substances from *S. chinensis* fruits. The active substances in *S. chinensis* fruit were extracted using the ethanol extraction method. *S. chinensis* fruit powder (100 g) was weighed and placed in a conical flask. The active substances were extracted for thrice using 70% ethanol at a material to liquid ratio of 1:20, each time for 60 min, and the filtrates were combined. The solvent was subsequently extracted twice (4 h each time) using 1:20 petroleum/ether and degreased. After extraction and separation, the ethanol layer was retained, and *S. chinensis* fruit extract was obtained through rotation and steaming at 60 ℃ in a water bath until no ethanol taste and/or smell remained. Finally, the extract was weighed.

#### Effects of *S. chinensis* fruit extract on *A. alternata* growth in vitro

*Alternaria alternate* was activated and inoculated on a potato dextrose agar (PDA) medium and incubated at 28 ℃ for 72 h. Antifungal experiments were performed using the mycelial growth rate method. The *S. chinensis* fruit extract was used as a sample to prepare the PDA culture medium with concentration gradients of 0, 500, 1000, 2000, 4000, and 8000 mg/L. After pouring it into a culture dish and allowing it to cool and solidify, activated *A. alternata* with a diameter of 6 mm was introduced an incubated at 28 ℃ for 72 h. The colony diameter was measured using the crossover method, and the inhibitory effect was calculated. Each measurement was performed in triplicate. The virulence regression equation was generated using Microsoft Excel 2021 software. An independent regression equation was generated using the logarithm of concentration as the abscissa and the probability value of the inhibitory rate as the ordinate.

### Detection of compound composition in *S. chinensis* extract

#### Compound extraction

The *S. chinensis* extract was accurately weighed into a 2 mL centrifuge tube, and 600 µL methanol (1:3 w/v) containing 2-chloro-l-phenylalanine (4 ppm) was added. The mixture was swirled for 30 s with glass beads (100 mg) in a tissue grinder, and grinding was conducted at 60 Hz for 90 s. Ultrasonication was performed for 15 min, followed by centrifugation at 12,000 rpm for 10 min at 4 ℃. The supernatant was passed through a 0.22 μm membrane filter, and the filtrate was transferred to a detection vial for LC–MS analysis^25^.

#### LC–MS detection

An ultra-high-performance liquid phase system equipped with a chromatographic column (2.1 × 150 mm, 1.8 µm) was employed for online detection. The flow rate was set to 0.25 mL/min and the column temperature was 40 ℃. An injection volume of 2 μL was used. In positive ion mode, the mobile phase comprised 0.1% formic acid-acetonitrile (C) and 0.1% formic acid–water (D); the following gradient elution was used: 0–1 min, 2% C; 1–9 min, 2–50% C; 9–12 min, 50–98% C; 12–13.5 min, 98% C; 13.5–14 min, 98–2% C; 14–20 min, 2% C. In negative ion mode, the mobile phase comprised of acetonitrile (A) and 5 mM ammonium formate in water (B); the following gradient elution was used: 0–1 min, 2% A; 1–9 min, 2–50% A; 9–12 min, 50–98% A; 12–13.5 min, 98% A; 13.5–14 min, 98–2% A; 14–17 min, 2% A^26^.

The MS conditions were as follows: mass spectrometer detector, electric spray ion source; data were collected in positive and negative ion modes. The positive and negative ion spray voltages were 3.50 kV and − 2.50 kV, respectively. The sheath gas was 30 arbitrary units (arb), whereas the auxiliary gas was 10 (arb). The capillary tube had a temperature of 325 ℃ and a resolution of 60,000 for a first-level full scan. The first-level ion scan range was m/z 100–1000. For second-level cracking, high-energy collision dissociation was used, with a collision voltage of 30% and a second-level resolution of 15,000. The first four ions of the collected signal were fragmented, and dynamic elimination was used to remove unnecessary MS/MS information^27^.

#### Compositional analysis

Identification of the compounds was initially confirmed based on their precise molecular weight (molecular weight error ≤ 30 ppm) and then based on the MS/MS fragment model, the Human Metabolome Database (http://www.hmdb.ca), METLIN (http://metlin.scripps.edu), Massbank (http://www.massbank.jp/), LipidMaps (http://www.lipidmaps.org), mzClond (https://www.mzcloud.org), and the confirmation notes obtained from the Panomic self-built standard database.

#### Analysis of the effect of benzocaine on *A. alternata *in vitro

*Alternaria alternata* was activated and inoculated on PDA medium and incubated at 28 ℃ for 72 h. Inhibitory assays were performed using the growth rate method^28^. A drug-loaded PDA medium with a specific concentration gradient was prepared using benzocaine as a sample. Activated *A. alternata* with a diameter of 6 mm was introduced into the medium after pouring it into a culture dish and allowing it to cool and solidify. Incubation was conducted at 28 ℃ for 72 h. The colony diameter was measured using a crossover method to determine the inhibitory effect. Each measurement was performed in triplicate. The virulence regression equation was generated.

### Analysis of the controlling effect of benzocaine on postharvest black spot on apple

#### Preventive

To assess the preventive effect, a 3 cm diameter circle was formed on the surface of Fuji apples by evenly spraying 50 μL of benzocaine (42.99 mg/L). Sterile water was sprayed as a control. *Alternaria alternata* with a diameter of 0.6 cm was inoculated at the centre of the sprayed circle. The apple was wrapped in plastic, and the lesion diameter was measured on the 7th day to calculate the preventive effect. This procedure was repeated in triplicate for each treatment group, resulting in 12 recorded apples.

#### Therapeutic effect

*Alternaria alternata* with a 0.6 cm diameter was inoculated on the surface of Fuji apples, which were subsequently wrapped in plastic and incubated at 28 ℃ for 12 h. 50 μL of benzocaine (42.99 mg/L) was sprayed on the inoculation site. The control group received the same amount of sterile water followed by wrapping in plastic. The lesion diameter was measured on the 7th day to calculate the treatment effect. This procedure was repeated in triplicate for each treatment group, resulting in 12 recorded apples.

#### Electron microscopy observation of the effect of benzocaine on the morphology of *A. alternata* mycelium

After 3 days of solid culture of *A. alternata*, a 0.6 cm diameter plate of fungi was obtained from the colony’s edge and placed at the centre of a 90 mm culture dish containing PDA solid culture medium. Benzocaine was added to the treatment groups at a concentration of 42.99 mg/L, but not the control group. The mycelium was fixed with 2.5% glutaraldehyde at 4 ℃ for 24 h, washed with 0.1 mol/L phosphoric acid buffer solution (pH 7.4) thrice, dehydrated with ethanol solution at 30%, 50%, 70%, 80%, 90%, and 95% and re-dehydrated thrice with anhydrous ethanol, each time for 8 min. Tert-butanol was used for replacement treatment twice, with each treatment lasting approximately 8 min. Subsequently, the sample was frozen at − 20 °C for 20 min, followed by drying using a critical dryer. Finally, gold ion sputtering was performed: one side of a carbon conductive tape was adhered to the plate, and the other side was adhered to the dried fungus body for ion sputtering of gold. Once the ion sputtering was completed, the sample was observed using SEM and images were captured for analysis.

#### Transcriptome sequencing and analysis

*Alternaria alternata* was inoculated on PDA medium for 72 h, and a ring of *A. alternata* was placed on 100 mL of potato dextrose broth (PDB) medium. Benzocaine was at the EC_50_ concentration (42.99 mg/L) in the treatment group, while the control group did not receive benzocaine. After 48 h of cultivation, the fungus body was washed with sterile water, frozen, and centrifuged. The fungi were packaged with liquid nitrogen quick-frozen dry ice and sent to Suzhou Panomic Biopharmaceutical Technology Co. for transcriptome sequencing analysis.

#### Real-time fluorescence quantitative PCR analysis

RNA extraction was performed using the Fungal RNA extraction kit. RNA purity and concentration were assessed using a microspectrophotometer. The quality of RNA was evaluated through 1% agarose gel electrophoresis. The qualified RNA was reverse transcribed into cDNA using a third-generation reverse transcription premix. β-Actin was selected as the internal reference gene, and primers were designed using Primer Express 5.0 software (Applied Biosystems). Real-time fluorescence quantitative PCR analysis was performed using the S antibody dye method and quantitative PCR premix. Specific experiments were performed, and relative quantitative results were analysed following the steps described in the kit^29^. The primer sequences were as follows: TRINITY DN2890_c1_g1 forward: 5′-AGTCTGGCTACAACCTGCTT-3′ and reverse: 5′-TCTTGGATCTGCAGCTTCTC-3′, TRINITY DN1846_c0_g1 forward: 5′-AGAAACTCAAGCCTTTGGAG-3′ and reverse: 5′-TTCGCTTTTGCAGTTCTATC-3′, TRINITY DN2931_c0_g1 forward: 5′-AACAACGACCAAGAAAGTGA-3′ and reverse: 5′-AACCAGACTTCGGTGAATTT-3′, TRINITY_DN384_c0_g1 forward: 5′-CTTCGCGGCTCTTACTAAA-3′ and reverse: 5′-CTAATATGCGAGCGACCA-3′, TRINITY_DN1071_c0_g1 forward: 5′-CTTTAGCAGATCCAACATCG-3′ and reverse: 5′-CCTGGTACGAGATGAAGAAGT-3′, TRINITY_DN5997_c0_g1 forward: 5′-GCGAGATTCTTGATGTTGAG-3′ and reverse: 5′-ATGTTGCTCTCGAGATTCCT-3′, TRINITY_DN3423_c0_g1 forward: 5′-CTACGACGCTTATGAGTTCC-3′ and reverse: 5′-GTGTCTCGAGGATCTTCTCA-3′, TRINITY_DN4625_c0_g1 forward: 5′-CGTTAAGAAGCAACCAATCA-3′ and reverse: 5′-GAGCGAGTGGTACTTTTCCT-3′, Β-actin forward: 5´-ACTTTCAACGTTCCAGCCTTC-3′ and reverse: 5′-CGTAAATTGGAACGACGACGTGAGTA-3′.

#### Metabolome analysis

*Alternaria alternata* was inoculated on PDA medium for 72 h, and a ring of *A. alternata* was placed on 100 mL of PDB medium. Benzocaine was at the EC_50_ concentration in the treatment group, while the control group did not receive benzocaine. After 48 h of cultivation, the fungus body was washed with sterile water, frozen, and centrifuged. Subsequently the fungi were packaged with liquid nitrogen quick-frozen dry ice and sent to Suzhou Panomic Biopharmaceutical Technology Co. for metabolome analysis.

### Supplementary Information

Supplementary Tables.

## Data Availability

The date and metadata used to produce the results of this study are publicly available in National Center for Biotechnology Information (NCBI) at https://www.ncbi.nlm.nih.gov/bioproject/?term=PRJNA983094 and Metabolomics Experiments and Derived Information (EMBL-EBI) at https://www.ebi.ac.uk/metabolights/MTBLS9699.
