# Supplementary material for: Inhibitory effect of benzocaine from Schisandra chinensis on Alternaria alternata
Source: Sci Rep. 2024 Mar 20;14:6691. doi: 10.1038/s41598-024-57237-1 (PMC10954763; doi:10.1038/s41598-024-57237-1)
Supplement: Supplementary file 1 — Supplementary Tables. [file 41598_2024_57237_MOESM1_ESM.docx]

**Inhibitory** **effect** **of** **benzocaine** **from** ***Schisandra*** ***chinensis*** **on**

***Alternaria*** ***alternata***

**Lin** **Fang** **Long1#,** **Qi** **Fang** **Zhao1#,** **Fu** **Long** **Zhang2,** **Ran** **Tang1,** **Jia** **Bao** **Wei1,** **Shan** **Guan1** ，**Yan** **Chen1***

**Supplementary** **Information**

| Supplementary Data 1  **Table 1.**  **Inhibitory** **activity** **of** ***S.*** ***chinensis*** **fruit** **extract** **on** **the** **growth** **of** ***A.*** ***alternata*** **mycelium** | | | | | | |
| --- | --- | --- | --- | --- | --- | --- |
| *S.* *Chinensis* fruit  extract concentration (mg/L) |  | colony diameter (mm) | Inhibi tory (%) | Toxicity  regression  equation (y=) | R2 | EC50 (mg/L) |
| 0  500  1000  2000  4000  8000 |  | 80  71.5  57  42  28  12 | 0  11.5  31.1  51.4  70.3  91.9 | 2.0674x + 4.4323 | 0.991 | 1882.00 (1748-2070) |

Supplementary Data 2

**Table 2.**

**Compounds** **in** ***S.*** ***chinensis*** **extract** **accounting** **for** **more** **than** **1%**

| Relative content ranking | Compound name | Molecular formula | Proportion |
| --- | --- | --- | --- |
| 1 | *P*elargonic acid | C9H18O2 | 18.93% |
| 2 | Benzocaine | C9H11NO2 | 14.19% |
| 3 | p-*O*ctopamine | C8H11NO2 | 5.16% |
| 4 | Acetylcholine chloride | C7H16NO2Cl | 4.38% |
| 5 | Schisandrin | C24H32O7 | 4. 19% |
| 6 | Pipecolic acid | C6H11NO2 | 3.65% |
| 7 | Melibiose | C12H22O11 | 3.29% |
| 8 | 3-Hydroxymethylglutaric acid | C6H10O5 | 3.23% |
| 9 | Myo-Inositol | C6H12O6 | 2.91% |
| 10 | Besigomsin | C23H28O7 | 2.41% |
| 11 | Citric acid | C6H8O7 | 2.41% |
| 12 | L-Malic acid | C4H6O5 | 2.36% |
| 13 | 4-Hydroxybenzoic acid | C7H6O3 | 2.33% |
| 14 | D-2-Hydroxyglutaric acid | C5H8O5 | 2.12% |
| 15 | D-Psicose | C6H12O6 | 2.07% |
| 16 | Galactosylglycerol | C9H18O8 | 1.96% |
| 17 | beta-Lactose | C12H22O11 | 1.79% |
| 18 | Glucosamine | C6H13NO5 | 1.43% |
| 19 | Pyroglutamic acid | C5H7NO3 | 1.31% |
| 20 | Maleic acid | C4H4O4 | 1.21% |
| 21 | Taurine | C2H7NO3 S | 1. 17% |
| 22 | 5-Aminopentanoic acid | C5H11NO2 | 1.03% |

Supplementary Data 3

**Table 3.**

**Inhibitory** **activity** **of** **benzocaine** **against** ***A.*** ***alternata*** **mycelial** **growth**

| Concentration (mg/L) | Antifungal rate (%) | Toxicity regression equation (y=) |  | R2 | EC50 (mg/L) |
| --- | --- | --- | --- | --- | --- |
| 0  25  50  100  200  400 | 0  39.2  50  71.6  78.4  89.2 | 1.2652x +  6.7291 |  | 0.985 | 42.99 (21.29-50.48) |

Supplementary Data 5

**Table** **4.**

**Verification** **results** **of** **differentially** **expressed** **genes** **in** **the** **transcriptome**

| Gene Number | log2FC | |
| --- | --- | --- |
|  | qPCR | Transcriptome |
| TRINITY_DN2890_c1_g1 | –5.465 | –8.247 |
| TRINITY_DN1846_c0_g1 | –4.016 | –6.276 |
| TRINITY_DN2931_c0_g1 | –2.869 | –5.155 |
| TRINITY_DN384_c0_g1 | –2.697 | –5.079 |
| TRINITY_DN1071_c0_g1 | 1.850 | 4.456 |
| TRINITY_DN5997_c0_g1 | 2.521 | 5.118 |
| TRINITY_DN3423_c0_g1 | 4.415 | 7.311 |
| TRINITY_DN4625_c0_g1 | 1.115 | 3.776 |
